# Development of Possible Next Line of Systemic Therapies for Gemcitabine-Resistant Biliary Tract Cancers: A Perspective from Clinical Trials

**DOI:** 10.3390/biom11010097

**Published:** 2021-01-13

**Authors:** Nai-Jung Chiang, Li-Tzong Chen, Yan-Shen Shan, Chun-Nan Yeh, Ming-Huang Chen

**Affiliations:** 1 National Institute of Cancer Research, National Health Research Institutes, Tainan 704, Taiwan; njchiang@nhri.org.tw (N.-J.C.); leochen@nhri.org.tw (L.-T.C.); 2 Department of Oncology, National Cheng Kung University Hospital, College of Medicine, National Cheng Kung University, Tainan 704, Taiwan; 3 Department of Internal Medicine, Kaohsiung Medical University Hospital and Kaohsiung Medical University, Kaohsiung 807, Taiwan; 4 Institute of Clinical Medicine, College of Medicine, National Cheng Kung University, Tainan 704, Taiwan; ysshan@mail.ncku.edu.tw; 5 Department of Surgery, National Cheng Kung University Hospital, Tainan 704, Taiwan; 6 Department of General Surgery and Liver Research Center, Chang Gung Memorial Hospital, Linkou Branch, Chang Gung University, Taoyuan 333, Taiwan; 7 Center for Immuno-Oncology, Department of Oncology, Taipei Veterans General Hospital, Taipei 112, Taiwan; 8 School of Medicine, National Yang Ming University, Taipei 112, Taiwan

**Keywords:** biliary tract cancer, next-line therapies, clinical trials

## Abstract

Biliary tract cancer (BTC) compromises a heterogenous group of tumors with poor prognoses. Curative surgery remains the first choice for localized disease; however, most BTC patients have had unresectable or metastatic disease. The gold standard therapy for these patients is chemotherapy with gemcitabine and cisplatin. There are no consensus guidelines for standard treatment in a second-line setting, although the data of the ABC-06 trial showed a slight survival benefit from oxaliplatin and 5-fluorouracil combination chemotherapy. Recent progress in comprehensive genomic profiling for advanced BTC (ABTC) has helped to clarify tumorigenesis and facilitate the coming era of precision medicine. Generally, targeted agents fail to show significant clinical benefits in unselected populations. Only fibroblast growth factor receptor 2 (*FGFR2*) fusion and isocitrate dehydrogenase (*IDH*)- and *BRAF* mutation-enriched populations have survival benefits from the corresponding inhibitors. Several interesting targeted agents for monotherapies or combination therapies with other compounds are currently ongoing or recruiting. Here, we review the published data from clinical trials of second-line therapies after the failure of gemcitabine-based chemotherapy in ABTC. The results were stratified by different genetic alternations, as well as by chemotherapy, targeted therapy and immunotherapy.

## 1. Introduction

Biliary tract cancer (BTC), including intrahepatic cholangiocarcinoma (IHCC), extrahepatic cholangiocarcinoma (EHCC), ampulla of Vater cancer (AVC) and gallbladder cancer (GBC), accounts for 3% of all gastrointestinal malignancies. Among them, IHCC is the second most common type of primary liver cancer. Historically, cholangiocarcinoma consisted of IHCC and EHCC but not GBC or AVC [1]. The incidence of BTC is increasing globally and is generally higher in Asian countries than in Western countries, especially IHCC in Thailand, China and South Korea [2,3]. In general, incidence of IHCC is higher than that of EHCC in Asian countries, while the opposite occurs in Western countries [4]. There are some identified risk factors for cholangiocarcinoma, such as parasite infection, primary sclerosing cholangitis, cholelithiasis, bile duct cyst and hepatitis B and C, which commonly induce chronic inflammation and injury to the biliary epithelium [5]. In addition, chronic liver disease resulting from metabolic syndrome and non-alcoholic steatohepatitis is emerging as a new risk factor [6]. Recently, a nationwide long-term cohort study in Taiwan showed that nucleos(t)ide analogue therapy could significantly reduce IHCC risk in patients with chronic hepatitis B, thereby offering a potentially preventive method for IHCC development [7]. For early-stage disease, complete surgical resection remains the only potentially curative therapy. However, approximately 60–70% of advanced BTC (ABTC) is diagnosed at an advanced stage and systemic therapy is considered to be the standard treatment.

Several prospective clinical trials have demonstrated an improvement in the median overall survival (OS) using combination chemotherapy for ABTC. First, pivotal phase III (ABC-02) and Japanese bridging phase II (BT-22) studies established the role of gemcitabine and cisplatin (GC) in first-line chemotherapy for patients with ABTC, with a median OS of 11 months [8,9]. Second, a randomized phase III JCOG 1113 study showed the non-inferiority of gemcitabine and S-1 (GS) to GC with a numerical median OS (15.1 months vs. 13.4 months), which met pre-planned statistical design [10]. Based on these results, GS can be considered an alternative regimen for ABTC patients who would not be tolerant to vigorous hydration or cisplatin-related toxicities. Generally, ABTC patients receiving GS treatment had a median OS of 12.5–15.1 months [10,11]. Recently, a triplet regimen of GC plus S-1 (GCS) showed a significantly higher response rate (42% vs. 15%) and a longer median OS (13.5 vs. 12.6 months; one-year OS rate: 59.4% vs. 53.7%; hazard ratio (HR) 0.79, *p* = 0.046) compared to GC doublet therapy in a phase III KHBO1401-MITSUBA study [2]. The statistical design assumed a one-year OS rate of 43% in GC and of 55% in GCS (one-sided α 0.05, and power 0.8). GS or GCS regimens may be considered for eligible Asian patients as a first-line treatment. Notably, there is a discrepancy in the planned treatment duration between Western and Asian studies, with 24 weeks in the ABC-02 study [9], 48 weeks in the BT-22 study [8] and continuous treatment until progression or intolerance in GS/GCS studies [2,10,11].

After progression to first-line chemotherapy, only 15–35% of patients were able to receive second-line treatment in Western studies [3,12,13], and up to 75–82% were able to receive such treatment in Japanese trials. [8,10]. The most common second-line chemotherapy in Japan was S-1 with or without gemcitabine after the failure of GC treatment and GC after the failure of GS treatment [10]. Patients who had available and effective second-line treatment survived longer and had a higher quality of life [14]. It is necessary to explore suitable subsequent therapies for patients with ABTC refractory to gemcitabine-based chemotherapy.

The recent whole-exome and targeted sequencing of BTC not only confirmed the frequent mutations in well-known genes including *TP53*, *KRAS* and *IDH1/2* but also revealed mutations in novel chromatin remodelling-associated genes, such as *BAP1*, *ARID1A* and *PBRM1* [15]. In addition, these studies have also identified new, recurrent driver genetic alternations in cholangiocarcinoma, such as *FGFR2* fusion and *IDH1* mutations that are potentially actionable with available pan-FGFR inhibitors and IDH1 inhibitors [16,17,18]. Notably, there are significant differences in the frequency of mutated genes in IHCC, with or without liver fluke (Opisthorchis viverrini, Ov), or hepatitis B virus infections, reflecting the impact of the causal agents on cholangiocarcinogenesis. Compared to Ov-related CCAs, mutations in *BAP1*, *IDH1* and *IDH2* were more frequently observed in non-Ov-related CCAs [15,19]. There is also a genomic heterogeneity between Asian and Western IHCCs [20]. The frequency of *KMT2C*, *BRCA1/2* and *DDR2* in Asian cohorts was significantly higher compared to that among Western patients. Moreover, a higher tumor mutation burden (TMB) and DNA repair mutation frequency were also observed in Asian studies.

A better understanding of comprehensive genetic profiling with matched effective therapeutic agents has facilitated hope in personalized treatment for BTC. Since BTC encompasses a variety of entities, we should not just treat BTCs as a whole after the failure with systemic chemotherapy. In the era of precision medicine, it is important to identify driver mutations or targetable alternations for the guidance of targeted therapy and immunotherapy in specific subpopulations with ABTC. The current review article summarizes the available results from clinical trials for second-line treatment in advanced and metastatic BTC, including published data and major active ongoing and recruiting trials. The treatments involve chemotherapy, targeted agents and immunotherapy.

## 2. Chemotherapy

Before 2019, only some retrospective small-scale prospective studies and meta-analyses had reviewed the efficacy of second-line chemotherapy. Two systemic reviews of 761 and 1391 patients showed that the response rate for the use of second-line therapy was only 7.7%, with a median progression-free survival (PFS) of 2.6–3.2 months and a median OS of 6.5–7.2 months [14,21]. Combined treatment was not superior to the use of a single agent as second-line treatment for ABTC in terms of therapeutic efficacy [21]. Recently, the randomized, open label, phase III study of ABC-06 compared oxaliplatin and 5-fluorouracil/leucovorin (FOFLOX) for 12 cycles plus an active symptomatic control (ASC) to ASC alone in 162 patients who had progressed after first-line GC treatment. For a hypothesized HR of 0.63 in OS benefit, 148 events were required (80% power; 5% two-sided α). Patients receiving FOLFOX presented a statistical but modest improvement of median OS compared to those with ASC (6.2 vs. 5.3 months; HR 0.69, *p* = 0.031). Grade III/IV treatment-related toxicities were more frequent in the FOFLOX arm than those in the ASC arm (59% vs. 39%), including fatigue, neutropenia and infection [22]. While the survival benefit of FOLFOX was limited, FOLFOX is still currently defined as the standard second-line chemotherapy for ABTC patients who failed GC treatment. The limitation of this study was using ASC as the control arm but not using 5-fluorouracil monotherapy in clinical practice [23], highlighting the unmet medical need for later therapeutic choices for this disease. Two randomized phase II trials of FOLFOX vs. irinotecan and 5-fluorouracil/leucovorin (FOLFIRI, NCT03464968) and liposomal irinotecan plus 5-fluorouracil vs. 5-fluorouracil (NCT03043547) are ongoing to apply chemotherapy as the control arm.

A phase II trial used S-1 monotherapy (40 mg/m^2^ twice daily for 28 days, followed by 14 day-off) in BTC patients who failed to Gemcitabine-based chemotherapy [24]. The efficacies of an objective response rate (ORR) of 7.5%, a median PFS of 2.5 months and a median OS of 6.8 months were noted in 40 patients. For the S-1 plus gemcitabine regimen, one phase II study for ABTC (*n* = 38) showed a partial response in six patients (15.8%) with a median PFS and OS of 5.8 and 15.9 months, respectively [25]. Thus, S-1 became one of the options for second-line treatment in the Asian BTC population after failure of platinum-based therapy.

To improve therapeutic efficacy, more potent combination chemotherapy and in combination with targeted agents were also tested, including irinotecan and oxaliplatin plus 5-fluorouracil/leucovorin (FOLFIRINOX), and the application of capecitabine, irinotecan and gemcitabine plus bevacizumab [26]. In a single-arm phase II trial, 30 patients were enrolled who had disease progression or intolerance under GC treatment benefited from FOLFIRINOX treatment, with a median PFS and OS of 6.2 and 10.7 months, respectively [27].

Nab-paclitaxel in combination with gemcitabine has been applied as the first-line treatment for metastatic pancreatic cancer. NAP-CAPABIL trial is a pilot study of nab-paclitaxel in combination with capecitabine as second line treatment of ABTC. Ten patients were enrolled with a median PFS and OS of 5.7 and 12.1 months respectively [28].

Systemic chemotherapy was the most common second-line treatment for ABTC patients who were refractory to gemcitabine, if no druggable genetic mutations or available targeted therapy or immunotherapy.

## 3. Targeted Therapy

To appropriately choose targeted therapies for ABTC patients majorly depends on the results of in-depth sequencing or next-generation sequencing. The common genetic mutations vary between IHCC, EHCC and GBC, with *IDH* mutation and *FGFR* rearrangement in IHCC and *HER2* aberrations in EHCC and GBC [29]. Currently, the successful clinical trials of targeted therapy in ABTC were mostly biomarker-driven.

### 3.1. FGFR2 Fusions

In IHCC, the aberrant activation of the FGFR signaling pathway leading to tumor cell migration stands for approximately 15 to 20% of patients, with the most common genetic alternation being *FGFR2* fusion [30]. A much lower detection rate of 2% was reported in a Chinese report [31]. Notably, the IHCC patients with *FGFR2* fusions have been frequently associated with several clinical features, such as female, younger age, early stage and better survival [32]. Co-existing *TP53* mutation and *CDKN2A/B* loss were correlated with a shorter median OS [33]. Recently, multiple pan-FGFR tyrosine kinase inhibitors (TKIs), such as pemigatinib (INCB054828), infigratinib (BGJ398), futibatinib (TAS-120), derazantinib (ARQ087), erdafitinib (JNJ-42756493) and rogoratinib (BAY1163877), have been applied for gemcitabine-refractory IHCC with *FGFR* genetic alternations. In this paper, we briefly summarize some of these completed studies.

There are two kinds of FGFR inhibitors divided by the efficacy to inhibit the FGFR4 activity. Some FGFR inhibitors selectively target FGFR1-3 but relatively serve as weak inhibitors to the FGFR4 activity, such as pemigatinib with additional vascular endothelial growth factor receptor 2 (VEGFR2) inhibition, and infigratinib. The others include futibatinib, derazantinib (with additional inhibition to RET, platelet-derived growth factor receptors, KIT and SRC) and erdafitinib with stronger activity to FGFR4 inhibition compared to the former group. Patients with *FGFR2* fusions or translocations had more benefit with a higher ORR (18.8–35.5%) under the treatment of FGFR inhibitors, compared to those with *FGFR2* mutation/amplification or other FGF/FGFR alternations [17].

In a FIGHT-202 study, patients had treatment-refractory advanced or metastatic cholangiocarcinoma harboring *FGFR2* gene fusion or rearrangement and took oral pemigatinib. Total 107 patients had an ORR of 35.5%, a DCR of 82% and a median PFS of 6.9 months. The median duration of response (DOR) was 9.1 months. The response persisted longer than six months in 24 of the 38 (63%) patients and longer than 12 months in seven (18%) patients. Hyperphosphatemia was the most common all-grade adverse event (AE) up to 60%, followed by arthralgia, stomatitis, diarrhea and fatigue [34]. Based on the promising result, the Food and Drug Administration (FDA) granted accelerated approval to pemigatinib (PEMAZYRE, Incyte Corporation, Wilmington, DE, USA) for the treatment of patients with previously treated cholangiocarcinoma with *FGFR2* fusion or other rearrangements in April 2020 [35]. Moreover, FoundationOne^®^ CDX (Foundation Medicine, Inc., Cambridge, MA, USA) was also approved by the FDA as a companion diagnostic for patient selection.

The other selective-FGFR inhibitor, BGJ398, showed meaningful efficacy in IHCC patients with *FGFR* alternations who failed under previous chemotherapy, with 14.8% ORR (18.8% in *FGFR2* fusion group, *n* = 48), 75% DCR and a median PFS 5.8 months among 61 patients. The most frequent treatment-related AEs are similar to those of previous items under pemigatinib treatment, except for palmar-plantar erythrodysesthesia [36]. The update efficacy in the expanded *FGFR2*-fusion population (*n* = 71) showed a confirmed ORR of 26.9%, a median PFS of 6.8 months and a median OS of 12.5 months [37]. Derazantinib (ARQ087), a multi-kinase inhibitor with potent activity against FGFR1-3 kinases and an additional inhibition to RET, platelet-derived growth factor receptors (PDGFR), KIT and SRC, was tested in treatment-refractory IHCC patients with *FGFR2* fusions in a phase I/II trial [38]. In the cohort of 29 patients, the results showed an ORR of 21%, a DCR of 83% and a median PFS of 5.7 months. Among them, two patients were treatment-naïve and others were refractory to at least one prior chemotherapy.

TAS-120 inhibits the kinase activity of FGFR1-4 irreversibly and highly potent, which may overcome some drug resistance from ATP-competitive FGFR inhibitors, such as BGJ398 [39]. In the dose-escalation phase I TAS120 study (16, 20 and 24 mg QD continuously), 45 IHCC patients harboring FGF/FGFR aberrations who received prior systemic therapies were enrolled. Of the 28 patients with *FGFR2* gene fusions, the ORR and DCR were 25% and 79%, respectively. Of the 13 patients who had received prior FGFR inhibitors, four (three with *FGFR2* gene fusions and one with *FGFR2* amplification) had confirmed PR [40]. Further trials are currently ongoing in patients with advanced solid tumors harboring FGF/FGFR aberrations, including cholangiocarcinoma (NCT02052778).

There are still many ongoing phase I and II trials on FGFR inhibitors in pretreated *FGFR*-fusion IHCC, such as E7090 (NCT04238715), CPL304110 (NCT04149691), EOC317 (NCT03583125) and INCB062079 (NCT03144661). Despite the evidence of the therapeutic efficacy of FGFR inhibitors, almost all patients eventually developed acquired resistance. This result leads to the development of innovative therapeutic strategies to overcome acquired drug resistance [16]. The common grade 3/4 AEs of FGFR inhibitors include hyperphosphatemia (22–29%), hypophosphatemia (7–14%), hyponatremia (8%), mucositis/stomatitis (7–18%), palmar-plantar erythrodysesthesia (5%), asthenia (6%) and abnormal liver function tests (6%) [36,38,40,41,42]. Hyperphosphatemia is an on-target AE related to inhibition of FGF23, which regulates the renal excretion of phosphorus and bone absorption [43,44,45]. On the contrary, hypophosphatemia is possibly related to overcorrection of hyperphosphatemia by dietary restriction and phosphate binders [37,42].

### 3.2. IDH1/2 Mutations and PARP

Somatic point mutations in *IDH1* and *IDH2* result in a gain-of-function for cancer cells, followed by the accumulation and secretion of the oncometabolite, D-2-hydroxyglutarate (2-HG) [46]. The accumulated 2-HG may inhibit specific α-KG–dependent dioxygenases and participate in tumorigenesis, including signal transduction, extracellular matrix maturation and epigenetic regulation [47]. *IDH1/2* mutations are primarily reported in 19–36% of IHCC [48]. However, the prognostic implication of *IDH1* mutation remained no conclusion [49]. Ivosidenib is an IDH1 inhibitor approved in the USA for patients with mutant *IDH1* (*mIDH1*) acute myeloid leukaemia. The ClarIDHy trial was a randomized, double-blinded, placebo-controlled phase III study designed to evaluate the efficacy of ivosidenib (500 mg once daily) in previously pre-treated IHCC patients harboring *IDH1* mutations [50]. The primary endpoint was PFS by independent central review and *p* value < 0.05 was considered to be a statistical significance. Patients receiving ivosidenib had a longer PFS (2.7 vs. 1.4 months, HR 0.37, *p* < 0.0001) and a comparable OS (10.8 vs. 9.7 months) to those who received the placebo, with 57% of placebo group crossover to ivosidenib when progression. Patients receiving ivosidenib had a favorable safety profile without treatment-related deaths. This study shows the clinical benefit of ivosidenib in advanced, *IDH1*-mutant cholangiocarcinoma. There are several clinical trials ongoing to test the efficacy and safety of other IDH inhibitors, such as BYA143602, IDH305, FT21012 and AG-881 (NCT02481154, NCT02746081, NCT02381886 and NCT03684811).

Poly-adenosine diphosphate-ribose polymerase (PARP) inhibitors serve as therapeutic agents, especially for *BRCA*-related high-grade serous ovarian cancer and *BRCA*-mutation breast cancers [51], as well as for those with DNA damage repair (*DDR*) gene aberrations [52]. In 28% to 64% of patients with BTCs, the *DDR* gene alterations have been identified, including but not limited to, mutations in *BRCA1*, *BRCA2*, *ATM*, *ATR*, *BAP1*, *BARD1*, *BRIP1*, *CHEK2*, *FAM175A*, *GEN1*, *MLH1*, *MSH2*, *MSH6*, *MRE11A*, *RAD50*, *RAD51*, *RAD51C*, *RAD51D*, *NBN*, *PALB2*, *PMS2*, *FANCA*, *FANCD2* and *XRCC2* [53,54]. Patients with *DDR* genetic mutations had a significantly longer PFS and OS among the ABTC patients who received first-line platinum-containing chemotherapies [53]. *BRCA1/2* mutations occur in 1–7% of BTC patients [55,56], which frequently come from somatic origin [57]. *BRCA2* mutation carriers have a lifetime risk of around 5% to develop BTC [55]. There are two ongoing phase II trials of PARP inhibitor associated with BTC: Niraparib for *BAP1* and other DDR pathway deficient neoplasms (NCT03207347) and olaparib for metastatic BTC with somatic or germline *DDR* mutations after the failure of platinum (NCT04042831). So far, there are no recommendations to assess *BRCA1/2* or *DDR* mutations to routinely in BTC subpopulations. This analysis is very important to determine the real prevalence of *BRCA 1/2* and germline/somatic *DDR* mutations in BTCs, as well as the impact of PARP inhibitors on this subpopulation.

Interestingly, a recent study suggested that *IDH1*/*2* mutations induce a “BRCAness” phenotype because of an induced defect of homologous recombination [58]. The oncometabolite, 2-HG, results in a hypermethylated status through inhibiting DNA and protein demethylation. IDH inhibitors in combination with PARP inhibitors or demethylating agents may represent a potential strategy in *IDH*-mutated CCA [58,59]. There are many ongoing clinical trials on using the PARP inhibitor in refractory, *IDH*-mutant CCAs, such as olaparib monotherapy (NCT03212274), olaparib plus durvalumab (NCT03991832) and olaparib plus a small molecule inhibitor targeting ataxia telangiectasia and the Rad3 related protein, ceralasertib (NCT03878095).

## 4. EGFR/HER2

The overexpression of epidermal growth factor receptor (EGFR) is generally reported in BTC, with a frequency of 39–100% [60]. Many trials focused on EGFR inhibitors as monotherapies or in combination with chemotherapy, mostly under first-line setting [61]. A phase II trial of the EGFR TKI erlotinib in refractory BTC patients showed a six-month PFS rate of 17% and three patients achieved PR (a total 42 subjects) [62]. In another phase II study of erlotinib plus docetaxel on hepatocellular carcinoma and biliary cancers after the failure of one prior systemic therapy, the combination regimen met the endpoint of a 16-week PFS ≥ 30% but with a comparable OS to single-agent erlotinib [63]. Due to the negative results from the studies of EGFR antibodies as a first-line therapy, such as cetuximab and panitumumab, there have been limited subsequent studies on second- or later-lines of treatment. Response-predicting *EGFR* mutation to EGFR TKIs involving exons 18, 19 and 21 had ever been described in a cohort of 22 cholangiocarcinoma patients. Of them, 13.6% of patients (*n* = 3) harbored *EGFR* mutations with all exon 19 deletion [64]. In another study, 6 of 40 (15%) of BTC patients had response-predicting *EGFR* mutations and all were within exon 21 [65]. Due to little or no clinical efficacy from EGFR TKI, the role of *EGFR* mutations to predict the response from EGFR TKIs in BTC remained uncertainly.

Recently, a phase I trial of chimeric antigen receptor-modified T (CART)-EGFR cell immunotherapy conditioned with nab-paclitaxel and cyclophosphamide was tested for EGFR-positive advanced BTC [66]. Of the total 17 evaluable cases, one patient achieved complete remission, and 10 subjects got stable disease (SD) with a median PFS of four months and an acceptable safety profile, indicating that CART-EGFR cell therapy provides a new feasible approach for advanced BTCs.

The agents targeting the HER2 signaling pathway, especially *HER2* amplification, significantly benefited patients with breast cancers and gastric cancers [67,68]. In addition, HER2 amplification, as well as overexpression were reported in 5–15% of EHCC and GBC, but with fewer in IHCC [69]. In the MyPathway basket trial of trastuzumab plus pertuzumab for BTC harboring *HER2* amplification/overexpression and mutation after the failure of previous treatment, the ORR was 7.5% in the amplified patients and 33.3% in the *HER2* mutated population [70]. The reasons of higher ORR in *HER2* mutated population than that in amplificated/overexpressed population may be due to small case number, which needs further exploration. In an ongoing SUMMIT basket trial of a pan-HER TKI, the efficacy of neratinib is currently being assessed in solid tumors harboring *EGFR*, *HER2* or *HER3* mutations/amplification [71]. The ORR was 10% among the 20 patients in the preliminary results [72]. Another trial yielded a PR of 27%, and a DCR of 70% in 37 BTC patients receiving the pan-HER TKI, varlitinib, in addition to cytotoxic chemotherapy [73]. Another TKI targeting EGFR/HER2/HER4, lapatinib, has shown modest benefits in BTC [74]. Currently, some ongoing trials are evaluating the efficacy of HER2-targeted therapies in BTC as a second-line treatment. For example, the efficacy of varlitinib (V) plus capecitabine (C) versus capecitabine alone was tested for gemcitabine–refractory patients in the double blind phase II TreeTopp study [75]. A Hochberg procedure had been used to control the familywise type I error rate at the 10% level (one-sided). Overall, 127 patients were randomized (64 of V + C; 63 of C) considering the dual primary endpoints of ORR and PFS. The results showed that V + C was well tolerated but did not improve the ORR (9.4% vs. 4.8%, *p* = 0.42), median PFS (2.8 vs. 2.8 months; *p* = 0.63) or median OS (7.8 vs. 7.5 months; *p* = 0.66) compared to V in 2L advanced BTC. In the similar setting, another phase II trial is currently assessing the role of trastuzumab in combination with chemotherapy in previously treated HER2-positive metastatic carcinomas of the digestive system, including metastatic BTC (NCT03185988). A phase I study of HER2-targeted CART therapy was also conducted on ABTC and pancreatic adenocarcinoma. Four patients had SD after treatment and one subject had a PR lasting for 4.5 months [76]. Trastuzumab deruxtecan (DS-8201) is an antibody-drug conjugate composed of an anti-HER2 antibody, cleavable terapeptide-based linker and a topoisomerase I inhibitor, which showed durable response in a wide spectrum of cancer subtypes with HER2 positive in a phase I study [77]. A multicenter phase II HERB trial of DS-8201 for HER2-positive ABTC is currently ongoing with primary endpoint of ORR [78].

## 5. RAF/MEK

The mitogen-activated protein kinase (MAPK)/extracellular signal-regulated kinase (ERK) pathway, also known as the Ras-Raf-MEK-ERK pathway, can regulate the proliferation, differentiation and apoptosis after activation [79]. Raf family members (Raf-1, B-Raf, and A-Raf) are Ras effectors and upstream activators of the ERK pathway. Recent studies have revealed that the v-Raf murine sarcoma viral oncogene homolog B (*BRAF*) mutation rates are high in certain cancers and the most common is *BRAF V600E,* with variable mutation rates in different populations [80]. Such mutations have been found in 5% of BTC, and may be enriched in IHCC [81]. A phase II basket trial of Vemurafenib monotherapy used a BRAF kinase inhibitor in previously treated non-melanoma patients with *BRAF V600E* mutations [82]. The results yielded an ORR of 12% in eight enrolled cases with CCA. In a phase II trial of selmeitinib, a MEK1/2 inhibitor, total 28 patients with previously-treated BTC were enrolled [83]. The results yielded an ORR of 12%, with a median PFS and OS of 3.7 and 9.8 months, respectively.

Another highly selective inhibitor of MEK1/2, trametinib, was applied in a randomized phase II trial (SWOG S1310) and was compared to fluoropyrimidine, including 5-fluorouracil or capecitabine in refractory ABTC [84]. The study was terminated early because of a poor response in the trametinib arm. For the 24 patients in the trametinib arm, the ORR, median PFS and median OS were 8%, 1.4 months and 4.3 months, respectively, compared to those in the chemotherapy arm (10%, 3.3 months and 6.6 months). Patients with refractory BTC did not benefit from trametinib. Two phase Ib trials were to test binimetinib, a selective MEK1/2 inhibitor, in BTC patients [85,86]. One of the binimetinib monotherapy was applied to 28 CCA patients, 43% of whom had previous first-line treatment [85]. The results showed an ORR of 7.1% (1 CR and 1 PR) with mild and common AEs. Another study combined binimetinib and capecitabine in 34 CCA patients who failed to gemcitabine-based chemotherapy [86]. Seven patients (20.6%) had PR, with a median PFS and OS of 4.1 months and 7.8 months, respectively [86]. Furthermore, patients harboring mutations associated with the RAS/RAF/MEK/ERK pathway got a significantly better ORR (40.0% vs. 12.5%) and longer median PFS (5.4 vs. 3.5 months) than those with wild-type tumors.

Combining both BRAF and MEK inhibitors would help delay the MAPK-driven acquired resistance, leading a better outcome, and decrease the toxicities observed from the paradoxical MAPK pathway activation after treatment with BRAF inhibitors [87]. In the basket-designed ROAR study, dabrafenib (D) plus trametinib (T) was tested in nine different cohorts of 178 patients with rare malignancies harboring *BRAF V600E* mutations [88]. The final report among the 43 patients with refractory BTC showed an investigator-assessed ORR of 51%, a median PFS and median OS of nine months and 14 months, respectively. The results indicated that D + T have promising efficacy in patients with ABTC, as well as favorable safety profile [89].

## 6. c-MET

The proto-oncogene *c-MET* encodes the receptor tyrosine kinase for its ligand, the hepatocyte growth factor, which plays the development of carcinogenesis by promoting tumor invasion, angiogenesis and metastasis [90]. c-MET over-expression was reported in 11.7% and 16.2% of IHCC and EHCC, respectively, and was associated with a shorter recurrence-free survival after surgery [91]. Whether c-MET overexpression served as a prognostic biomarker or not remained inconclusive. Cabozantinib, a multi-kinase TKI targeting MET, was applied in a phase II study and showed limited activity in 19 previously treated CCA patients with a median PFS of 1.8 months and an median OS of 5.2 months [92]. In a phase I trial of an oral MET inhibitor, tivatinib (ARQ197) in combination with gemcitabine, 56 evaluable patients had an ORR of 20%. Among the right CCA patients, only one had PR [93].

## 7. PI3K/AKT/mTOR

The PI3K/AKT/mTOR pathway is a core regulator of cell metabolism, proliferation and survival, and is involved in BTC carcinogenesis and progression [94]. The aberrations in PI3K/AKT/mTOR signaling mainly consist of *PI3KCA* mutations/amplifications, PTEN loss, phosphorylated AKT or mTOR over-expression in BTC [95,96,97]. Several trials have applied to test the efficacy of PI3K/AKT/mTOR inhibitors in first- and second-line settings, but the results showed limited activity [98,99]. A phase II trial of MK-2206, an AKT selective inhibitor, in second-line treatment was terminated early [100]. Ultimately, eight CCA patients were enrolled with no responders, a median PFS of 1.7 months and a median OS of 3.5 months. For mTOR inhibitors, a phase II ITMO trial evaluated everolimus in 39 CCA patients after prior chemotherapy [101]. The results showed an ORR of 5.1%, with a median PFS and a median OS of 3.2 and 7.7 months, respectively. To achieve a better response from targeting the PI3K/AKT/mTOR pathway, it is important to explore the biomarkers for patient selection and determine the resistant mechanisms and co-existing drivers.

## 8. Anti-Angiogenesis Agents

Both angiogenesis and lymphangiogenesis play an important role in BTC tumorigenesis [102,103]. Several compounds related to vascular endothelial growth factor (VEGF) pathway have been tested in phase I and II trials of second- or later-line treatments, including monoclonal antibodies of bevacizumab and ramucirumab and TKIs of sorafenib, sunitinib, cediranib, regorafenib, selumetinib, lenvatinib and apatinib, as monotherapy or in combination with chemotherapy or other antitumor agents.

Bevacizumab plus chemotherapy has been applied in a phase II studies with capecitabine, irinotecan and gemcitabine for second-line treatment in patients with metastatic ABTC [26]. A total of 50 subjects were enrolled with an ORR of 6%, a median PFS of 3.6 months and a median OS 6.4 of months. For the anti- VEGFR2 human monoclonal antibody, ramucirumab, a phase II trial of monotherapy is ongoing (NCT02520141).

A phase II trial sorafenib was tested in 46 ABTC patients, in which 26 (56%) had failed to first-line treatment [104]. Sorafenib monotherapy presented a limited activity in ABTC, with an ORR of only 2%, a median PFS of 2.3 months and a median OS of 4.4 months. Sunitinib was tested in a phase II SUN-CK trial as second-line treatment in 53 patients with advanced IHCC [105]. The efficacy yielded an ORR of 15%, a median PFS of 5.2 months and a median OS of 9.6 months. A similar study design was also applied to a phase II trial of the Asian population [106]. Of the 56 patients, five subjects (8.9%) had PR, and 23 had SD, with a median time to tumor progression of 1.7 months, suggesting that sunitinib monotherapy had marginal efficacy against metastatic BTC.

Cediranib, an oral VEGFR inhibitor, has been used in combination with erlotinib, a multi-targeting tyrosine kinase inhibitor of VEGFR, FGFR, PDGFR and c-kit as a second-line treatment for patients with ABTCs [107]. A total of 20 patients will be enrolled. So far, eight patients have undergone at least one efficacy evaluation, of which two (25%) had PR and five showed SD (62.5%).

Regorafenib monotherapy was evaluated in a phase II trial of patients with chemotherapy-refractory BTC [108]. A total of 43 patients were enrolled. Of the 34 evaluable patients for tumor response, a partial response was achieved in five patients (11%) with a median PFS of 15.6 weeks and a median OS of 31.8 weeks. Grade 3 and 4 AEs were noted in 40% of patients. In another phase II trial, regorafenib was used with an identical study design in 39 ABTC patients after the failure of prior gemcitabine-based chemotherapy [109]. The ORR was 6%, with a median PFS and OS of 3.7 and 5.4 months, respectively. Overall, 72% of the patients experienced grade 3/4 AEs. Furthermore, a randomized double-blinded placebo-controlled phase II REACHIN trial used regorafenib after the failure of gemcitabine plus platinum-based regimens in advanced and metastatic BTC, with the primary endpoint of PFS [110]. No crossover was allowed. Assuming an improvement in median PFS from 1.5 months to 3 months with one-sided significance level of 0.1 and power of 80%, 38 PFS events will be needed. In total, 66 (33 per group) patients were included. The results showed that regorafenib provided a significantly longer median PFS (3.0 vs. 1.5 months, *p* = 0.004; HR 0.49) and a better SD rate (74% vs. 34%, *p* = 0.002) compared to the placebo group. However, the median OS was numerically comparable between the two groups (5.3 vs. 5.1 months, *p* = 0.28). Fourteen of 33 patients (42%) had a dose reduction in the regorafenib group [110]. Regorafenib significantly improved PFS and disease control as the second- or third-line setting in patients with refractory ABTC.

Selumetinib inhibiting the kinase activity of MEK1/2 was tested in a phase II study on patients with metastatic BTC. Of the 28 enrolled patients, 39% had received one prior systemic therapy. The results showed that three patients (12%) had a confirmed objective response, with a median PFS of 3.7 months and a median OS of 9.8 months. The most frequent toxicities were rash and xerostomia [83].

A phase II trial used lenvatinib monotherapy (24 mg/day) as the second-line treatment for ABTC [111]. Of the preliminary results in 26 patients, the ORR was 12%, with a median PFS and a median OS of 3.2 and 7.4 months, respectively. A trial using Apatinib, a TKI targeting VEGFR-2 from China as a second-line therapy in patients with refractory BTC is currently recruiting (NCT03144856).

## 9. NTRK

Neurotrophic tropomyosin receptor kinase (*NTRK*) gene fusions are oncogenic drivers involving either *NTRK1*, *NTRK2 or NTRK3* genes with corresponding neurotrophic receptors of TRKA, TRKB and TRKC, respectively [112]. *NTRK* fusions have recently emerged as interesting driver mutations for cancer treatment, which have been detected in adult and pediatric cancers [113]. In BTC, the reported percentage of *NTRK* fusions was 0.75% [114]. Larotrectinib (LOXO-101) and entrectinib (RDX-101) are first-generation, potent and highly selective pan-TRK inhibitors that were approved by the FDA, for pediatric and adult patients with refractory solid tumors harboring *NTRK* gene that progressed after standard treatments without suitable alternative treatments [115]. In three multicenter, open-label, single-arm clinical trials (LOXO-TRK-14001, SCOUT and NAVIGATE), 22% patients got complete response (CR) and 53% had PR, with an acceptable AE profile in 55 kinds of *NTRK*-positive malignancies [116]. Of these studies, two cases featured previously treated CCA and one case had PR. The efficacy of entrectinib from three clinical trials (ALKA-372-001, STARTRK-1 and STARTRK-2) yielded an ORR of 63.5% (95% CI, 51.5–74.4) and a CR of 7% with the most common ≥grade 3 treatment-related AEs being weight gain (7%), anemia (7%) and fatigue (6%) in the 74 *NTRK*-positive malignancies [117,118]. Only one case presented CCA and had PR after treatment [119]. Due to the exciting efficacy of NTRK inhibitors in agnostic tumors with *NTRK* fusion, testing BTC patients for *NTRK* aberrations is reasonable after the failure to first-line chemotherapy.

While patients with BTC receiving some targeted agents got PFS benefit compared to those receiving placebo, median OS did not show significant difference between two groups [50,108]. Furthermore, the control arm of targeted therapy-contained clinical trials was mostly placebo but not systemic chemotherapy, which served as an ethical issue actually [50,108]. Therefore, targeted therapy is still suggested as the second- or later-line treatment after the failure to first-line chemotherapy, even in subpopulation with specific genetic alternations. To move the next step of targeted therapy forward, relevant targets and adequate patient enrichment/selection by useful biomarkers are needed. Despite whole genome/exome and multi-omics data could provide more information, currently targeted gene sequencing panels still remained as a time-saving and effective tool for clinical use. Variable/feature selection methods that are particularly effective for pinpointing the important omics features associated with the prognostic outcomes [120,121]. Other disease phenotypes in cancer profiling studies should also be taken into consideration to broaden scope and add strength to this survey.

## 10. Immune Checkpoint Inhibitors

Immune checkpoint inhibitors (ICIs) have been widely applied in the treatment of several gastrointestinal malignancies, including esophageal squamous cell carcinoma, hepatocellular carcinoma and gastric adenocarcinoma [122]. Some immune biomarkers can potentially predict the therapeutic efficacy of ICIs, such as programmed death ligand 1 (PD-L1) protein expression, mismatch repair deficiency (dMMR)/microsatellite instability high (MSI-H) and tumor mutational burden (TMB) [123]. In a recent report, high PD-L1 expression was associated with a better response to pembrolizumab in patients with ABTC [124]. On May 2017, the Food and Drug Administration (FDA) approved pembrolizumab for the treatment of adult and pediatric patients with advanced, MSI-H or dMMR solid tumors that had failed to prior therapy [125]. TMB can be used to predict ICI efficacy and has become a useful biomarker in some cancer types to enrich patients who may get benefit from immunotherapy [126]. In June 2020, the FDA granted accelerated approval to pembrolizumab for patients who had advanced tumors with high TMB (≥10 mutations/megabase). The phase II KEYNOTE-158 trial has shown the robust activity of the anti-PD1 Ab pembrolizumab in previously treated patients with MSI-H/dMMR solid tumors. In total, 22 CCA patients had an ORR of 40.9%, a median PFS of 4.2 months and an OS of 24.3 months [127]. In the KEYNOTE-028 phase Ib trial of pembrolizumab monotherapy, 24 pre-treated patients (20 CCAs and 4 GBCs) with PD-L1 positive tumors showed an ORR of 13% and a median OS of 6.2 months [128]. More recently, 104 patients with pre-treated ABTC regardless of PDL1 expression were enrolled to receive pembrolizumab in the KEYNOTE-158 phase II trial [129]. The resulted showed the ORR was 5.8%, with a median PFS and OS of 2.0 and 7.4 months, respectively. In total, 60% of the patients with PD-L1 positive tumors had an ORR of 6.6%. Furthermore, a single arm phase II trial of pembrolizumab as a second-line treatment in patients with metastatic and refractory BTC, accompanied by the genomic analysis is currently ongoing (NCT03110328). For pembrolizumab combination therapy in pre-treated ABTC patients, trials of pembrolizumab plus the induction GM-CSF (NCT02703714) and pembrolizumab plus chemotherapy using capecitabine and oxaliplatin (NCT03111732) are currently ongoing.

Nivolumab, an anti-PD-1 agent, was first tested in a phase I study as monotherapy in Japanese patients who were refractory or intolerant to gemcitabine-based treatment regimens. Among the 30 enrolled patients, only one subject achieved PR (3.3%), with a median OS of 5.2 months [130]. A similar study design was also applied in a phase II trial of 54 BTC pre-treated patients [131]. The results showed an ORR of 22% by investigator-assessed review and 11% by central independent review, with a median PFS and OS of 3.7 and 14.2 months, respectively. All responders featured mismatch repair protein-proficient tumors.

Recently, an IO/IO combination of ipilimumab and nivolumab (nivolumab 3 mg/kg and ipilimumab 1 mg/kg every three weeks for four doses, followed by nivolumab 3 mg/kg every two weeks up to 96 weeks) in patients with ABTC was applied in a CA209-538 phase II study [132]. In total 39 patients with ABTC were enrolled and 33 experienced disease progression after one or more lines of therapy. The results showed that the ORR was 23% (*n*  =  9) with a DCR of 44% (*n*  =  17) and a median of PFS and OS of 2.9 months and 5.7 months, respectively. Immune-related AEs were reported in 49% of patients, with 15% experiencing grade 3 or 4 events. Interestingly, PARP inhibitors were able to upregulate PD-L1 expression and promote cancer-associated immunosuppression [133]. A phase II trial to combine nivolumab and the PARP inhibitor, rucaparib in patients with advanced or metastatic BTC following platinum therapy is ongoing (NCT 03639935).

A phase I trial studied the durvalumab, an anti-PD-L1 monoclonal Ab, as a monotherapy (D, *n* = 42) or plus tremelimumab (D + T, *n* = 65) in Asian pre-treated ABTC patients [134]. In the D cohort, two subjects had a PR (4.8%) and seven had a PR (10.8%) in the D + T cohort. The median OS was 8.1 (95% CI, 5.6–10.1) months and 10.1 (95% CI, 6.2–11.4) months for the D and D + T cohorts, respectively. Treatment-related AEs ≥ grade 3 were noted in 19% and 23% of patients in the D and D + T subpopulations. Both D alone and D + T combination therapies were tolerable without unexpected toxicities. Recently, a pilot study of D + T and radiation (XRT) for metastatic BTC that has progressed following one line of previous therapy enrolled 15 patients [135]. Of those who reached radiation (*n* = 12), the DCR was 33%, with a 17% PR and 8% CR. Treatment-related AEs ≥ grade 3 were seen in 9 out of 15 patients (60%).

Since the downstream crosstalk of the immune checkpoint pathway and VEGFR signaling may lead to a synergistic effects, this strategy has been applied to many malignancies [136], as well as ABTC. A phase I trial of pembrolizumab plus ramucirumab showed limited clinical activity in 26 previously treated metastatic BTCs, with an ORR of 4%, a median PFS of 1.6 months and a median OS of 6.4 months [137]. Another phase II trial of lenvatinib plus pembrolizumab or nivolumab in 14 IHCC patients who had failed to more than two prior treatments showed an ORR of 21.4% and a median PFS of 5.9 months [138]. Grade 3 of AEs occurred in 14% without grade 4 AEs. A phase II LEAP-005 trial (NCT03797326) is ongoing to test the combination of pembrolizumab plus lenvatinib in previously treated patients with solid malignancies, including BTC. Another recombinant anti-human PD-1 IgG4 monoclonal antibody, Toripalimab, in combination with lenvatinib in a phase II ABTC trial as a second-line treatment is ready to start in a phase II ABTC trial (NCT04211168).

Another kind of IO combination using the MEK inhibitor was applied in a phase II trial, atezolizumab (anti-PD-L1 Ab) as monotherapy or in combination with cobimetinib (21 days on/7 days off) in pre-treated ABTC [139]. The trial met its primary endpoint, which was compared between groups under the assumption of Cox proportional hazards. The median PFS in combination group was significantly longer than that in monotherapy group (3.7 vs. 1.9 months, *p* = 0.027). There was 1 PR (3.2%) and 13 SD (41.9%) in the combination arm, and 1 PR (2.9%) and 10 SD (29.4%) in the atezolizumab monotherapy arm.

Based on above previous literature review, anti-PD1 agents were only recommended in ABTC harboring MSI/dMMR and high TMB. For PDL1+ ABTC, ICIs alone or in combination other agents may serve one of therapeutic choices. Other predictive biomarkers are needed to predict the response of immunotherapy in ABTC.

## 11. Complementary Agents

Recently, complementary agents for the integrated management of cancer in several malignancies had been described, such as pancreatic cancer and colon cancer [140,141]. The major functions of most complementary agents in cancer treatment focus on the relief of cancer cachexia and fatigue, as well as in combination with cytotoxic agents to have synergistic effect. Racol^®^, an enteral nutrient formulated with omega-3 fatty acids, which may improve cancer cachexia was applied for 27 patients with unresectable pancreatic and BTC [142]. Skeletal muscle mass was significantly increased after the start of administration compared to that on baseline. Calcitriol, the active analogue of vitamin D which was reported to enhance the cytotoxicity of several anticancer agents, including gemcitabine [143]. A phase 1 study of gemcitabine in combination with escalating doses of weekly intravenously in patients with advanced cancers enrolled 44 patients, with one of GBC. The ORR was 4% with a DCR of 67% in patients who were pretreated with gemcitabine [144]. Other complementary agents are mostly in pre-clinical research and further clinical trials are needed to prove their efficacies.

## 12. Conclusions

**Figure 1:**
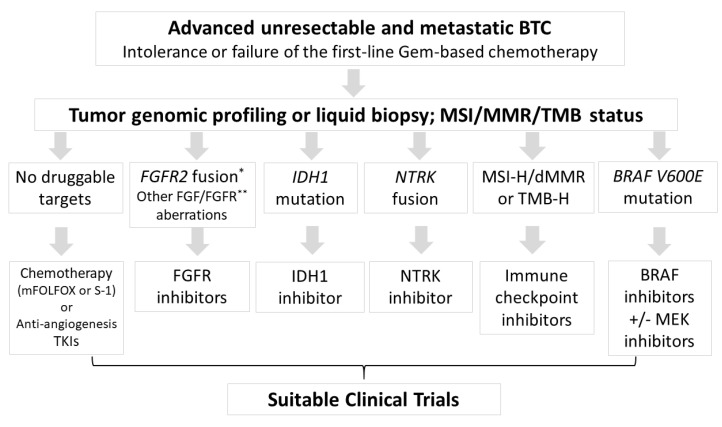
Flow chart for therapeutic choices after intolerance or failure to gemcitabine-based chemotherapy. According to the genetic alternations from tumor genomic profiling or liquid biopsy, some specific inhibitors were suggested in specific populations. MSI-H, microsatellite instability high; dMMR, deficiency of mismatch repair; TMB-H, tumor mutation burden high; TKI, tyrosine kinase inhibitors. * *FGFR2* fusion or rearrangement; ** such as mutation or amplification.

In Summary, cytotoxic chemotherapy remains the priority second-line treatment after the failure of gemcitabine-based chemotherapy in ABTC if no targetable genetic alternations can be identified. Currently, the driver mutations with effective inhibitors are limited only to *FGFR2* fusion, *IDH1*, *BRAF* mutations and potential immune checkpoint blockade, as shown in Table 1. Tumor agnostic therapies, such as pembrolizumab for MSI-H/dMMR status or the NTRK inhibitor for *NRTK* fusions, are applied in relatively small and specific populations (Figure 1). By optimizing personalized treatment, comprehensive genomic profiling from tumor tissue or liquid biopsies would become available more widely for drug selection. The exploration of novel targets with corresponding specific targeting agents is required to improve patient outcomes. The revolution of precision medicine in BTC is rapidly changing and multi-institutional collaboration is necessary for the completion of biomarker-driven clinical trials in this rare malignancy.

## Figures and Tables

**Table 1:** Selected representative trials of chemotherapy, targeted therapy and immunotherapy in advanced biliary tract cancer (ABTC).

Drug(s)	Study	Case (*n*)	Target	ORR (%)	SD (%)	PFS (mos)	OS (mos)
chemotherapy							
mFOLFOX vs. ASC [22]	ABC06 Phase III	162 (81 vs. 81)	-	4%	28%	4	6.2
S-1 [24]	Japan Phase II	40	-	7.5%	55%	2.5	6.8
FOLFIRINOX [27]	Phase II	30	-	10%	57%	6.2	10.7
Targeted therapy							
Pemigatinib [34]	FIGHT202Phase II	107 *	FGFR1-3 VEGFR2	35.5%	46.5%	6.9	21.1
Infigratinib (BGJ398) [36]	Phase II	71 *	FGFR1-3	26.9% ^&^	56.7%	6.8	12.5
Derazantinib (ARQ087) [38]	Phase I/II	30 *	FGFR1-3 RET, PDGFR, KIT, SRC	20.7%	62.1%	5.7	-
Fuibatinib (TAS-120) [39]	Phase I/II	28 *	FGFR1-4	25%	54%	-	-
Ivosidenib vs. placebo [50]	ClarIDHy Phase III	185 ** (124 vs. 61)	Mutant *IDH1*	2.4%	50.8%	2.7	10.8
Dabrafenib + trametinib [88]	ROAR Phase II	43 ***	*BRAF* *V600E*	51%	-	9	14
Regorafenib vs. placebo [108]	REACHIN Phase II	66(33 vs. 33)	VEGF1-3, PDGFR, FGFR	0%	74%	3	5.3
Immunotherapy							
Pembrolizumab [127]	KN-158 Phase II	104	PD-1	5.8%	-	2	7.4
Nivolumab [131]	Korea Phase II	54	PD-1	22% ^$^ 11% ^#^	-	3.7	14.2
Durvalumab + Tremelimumb [134]	Phase I	42 (D) 65 (D + T)	PD-L1 + CTLA-4	5% (D) 11% (D + T)	-	-	8.1 (D) 10.1 (T + D)

ABTC, advanced biliary tract cancer; ORR, objective response rate; SD, stable disease; PFS, progression-free survival; OS, overall survival; ASC, active symptomatic control; D, durvalumab; T, tremelimumab; vs., versus. * *FGFR2* translocations or fusions; ** *IDH1* mutations; *** *BRAF V600E* mutations; ^&^ confirmed ORR; ^$^ Investigator-assessed review; ^#^ central independent review.

## Data Availability

Not applicable.

## References

[1] Tariq N.U., McNamara M.G., Valle J.W. (2019). Biliary tract cancers: Current knowledge, clinical candidates and future challenges. Cancer Manag. Res..

[2] Banales J.M., Cardinale V., Carpino G., Marzioni M., Andersen J.B., Invernizzi P., Lind G.E., Folseraas T., Forbes S.J., Fouassier L. (2016). Cholangiocarcinoma: Current knowledge and future perspectives consensus statement from the European Network for the Study of Cholangiocarcinoma (ENS-CCA). Nat. Rev. Gastroenterol. Hepatol..

[3] Kirstein M.M., Vogel A. (2016). Epidemiology and Risk Factors of Cholangiocarcinoma. Visc. Med..

[4] Bragazzi M.C., Cardinale V., Carpino G., Venere R., Semeraro R., Gentile R., Gaudio E., Alvaro D. (2011). Cholangiocarcinoma: Epidemiology and risk factors. Transl. Gastrointest. Cancer.

[5] Pellino A., Loupakis F., Cadamuro M., Dadduzio V., Fassan M., Guido M., Cillo U., Indraccolo S., Fabris L. (2018). Precision medicine in cholangiocarcinoma. Transl. Gastroenterol. Hepatol..

[6] Lee T.Y., Hsu Y.C., Yu S.H., Lin J.T., Wu M.S., Wu C.Y. (2018). Effect of Nucleos(t)ide Analogue Therapy on Risk of Intrahepatic Cholangiocarcinoma in Patients With Chronic Hepatitis B. Clin Gastroenterol Hepatol..

[7] Okusaka T., Nakachi K., Fukutomi A., Mizuno N., Ohkawa S., Funakoshi A., Nagino M., Kondo S., Nagaoka S., Funai J. (2010). Gemcitabine alone or in combination with cisplatin in patients with biliary tract cancer: A comparative multicentre study in Japan. Br. J. Cancer.

[8] Valle J., Wasan H., Palmer D.H., Cunningham D., Anthoney A., Maraveyas A., Madhusudan S., Iveson T., Hughes S., Pereira S.P. (2010). Cisplatin plus gemcitabine versus gemcitabine for biliary tract cancer. N. Engl. J. Med..

[9] Morizane C., Okusaka T., Mizusawa J., Katayama H., Ueno M., Ikeda M., Ozaka M., Okano N., Sugimori K., Fukutomi A. (2019). Combination gemcitabine plus S-1 versus gemcitabine plus cisplatin for advanced/recurrent biliary tract cancer: The FUGA-BT (JCOG1113) randomized phase III clinical trial. Ann. Oncol..

[10] Morizane C., Okusaka T., Mizusawa J., Takashima A., Ueno M., Ikeda M., Hamamoto Y., Ishii H., Boku N., Furuse J. (2013). Randomized phase II study of gemcitabine plus S-1 versus S-1 in advanced biliary tract cancer: A Japan Clinical Oncology Group trial (JCOG 0805). Cancer Sci..

[11] Sakai D., Kanai M., Kobayashi S., Eguchi H., Baba H., Seo S., Taketomi A., Takayama T., Yamaue H., Ishioka C. (2018). Randomized phase III study of gemcitabine, cisplatin plus S-1 (GCS) versus gemcitabine, cisplatin (GC) for advanced biliary tract cancer (KHBO1401-MITSUBA). Ann. Oncol..

[12] Fornaro L., Cereda S., Aprile G., Di Girolamo S., Santini D., Silvestris N., Lonardi S., Leone F., Milella M., Vivaldi C. (2014). Multivariate prognostic factors analysis for second-line chemotherapy in advanced biliary tract cancer. Br. J. Cancer..

[13] Van Cutsem E., Kohne C.H., Hitre E., Zaluski J., Chang Chien C.R., Makhson A., D’Haens G., Pinter T., Lim R., Bodoky G. (2009). Cetuximab and chemotherapy as initial treatment for metastatic colorectal cancer. N. Engl. J. Med..

[14] Lamarca A., Hubner R.A., David Ryder W., Valle J.W. (2014). Second-line chemotherapy in advanced biliary cancer: A systematic review. Ann. Oncol..

[15] Wardell C.P., Fujita M., Yamada T., Simbolo M., Fassan M., Karlic R., Polak P., Kim J., Hatanaka Y., Maejima K. (2018). Genomic characterization of biliary tract cancers identifies driver genes and predisposing mutations. J. Hepatol..

[16] Krook M.A., Lenyo A., Wilberding M., Barker H., Dantuono M., Bailey K.M., Chen H.-Z., Reeser J.W., Wing M.R., Miya J. (2020). Efficacy of FGFR inhibitors and combination therapies for acquired resistance in FGFR2-fusion cholangiocarcinoma. Mol. Cancer Ther..

[17] Lamarca A., Barriuso J., McNamara M.G., Valle J.W. (2020). Molecular targeted therapies: Ready for prime time in biliary tract cancer. J. Hepatol..

[18] Rizvi S., Gores G.J. (2017). Emerging molecular therapeutic targets for cholangiocarcinoma. J. Hepatol..

[19] Jusakul A., Kongpetch S., Teh B.T. (2015). Genetics of Opisthorchis viverrini-related cholangiocarcinoma. Curr. Opin. Gastroenterol..

[20] Cao J., Hu J., Liu S., Meric-Bernstam F., Abdel-Wahab R., Xu J., Li Q., Yan M., Feng Y., Lin J. (2020). Intrahepatic Cholangiocarcinoma: Genomic Heterogeneity Between Eastern and Western Patients. JCO Precis. Oncol..

[21] Ying J., Chen J. (2019). Combination versus mono-therapy as salvage treatment for advanced biliary tract cancer: A comprehensive meta-analysis of published data. Crit. Rev. Oncol. Hematol..

[22] Lamarca A., Palmer D.H., Wasan H.S., Ross P.J., Ma Y.T., Arora A., Falk S., Gillmore R., Wadsley J., Patel K. (2019). ABC-06 | A randomised phase III, multi-centre, open-label study of active symptom control (ASC) alone or ASC with oxaliplatin/5-FU chemotherapy (ASC+mFOLFOX) for patients (pts) with locally advanced/metastatic biliary tract cancers (ABC) previously-treated with cisplatin/gemcitabine (CisGem) chemotherapy. J. Clin. Oncol..

[23] Brieau B., Dahan L., De Rycke Y., Boussaha T., Vasseur P., Tougeron D., Lecomte T., Coriat R., Bachet J.B., Claudez P. (2015). Second-line chemotherapy for advanced biliary tract cancer after failure of the gemcitabine-platinum combination: A large multicenter study by the Association des Gastro-Entérologues Oncologues. Cancer.

[24] Suzuki E., Ikeda M., Okusaka T., Nakamori S., Ohkawa S., Nagakawa T., Boku N., Yanagimoto H., Sato T., Furuse J. (2013). A multicenter phase II study of S-1 for gemcitabine-refractory biliary tract cancer. Cancer Chemother Pharm..

[25] Arima S., Shimizu K., Okamoto T., Toki M., Suzuki Y., Okano N., Naruge D., Kawai K., Kobayashi T., Kasuga A. (2017). A Multicenter Phase II Study of Gemcitabine plus S-1 Chemotherapy for Advanced Biliary Tract Cancer. Anticancer Res..

[26] Larsen F.O., Markussen A., Diness L.V., Nielsen D. (2018). Efficacy and Safety of Capecitabine, Irinotecan, Gemcitabine, and Bevacizumab as Second-Line Treatment in Advanced Biliary Tract Cancer: A Phase II Study. Oncology.

[27] Belkouz A., de Vos-Geelen J., Mathôt R.A.A., Eskens F.A.L.M., van Gulik T.M., van Oijen M.G.H., Punt C.J.A., Wilmink J.W., Klümpen H.-J. (2020). Efficacy and safety of FOLFIRINOX as salvage treatment in advanced biliary tract cancer: An open-label, single arm, phase 2 trial. Br. J. Cancer.

[28] Prager G.W., Kornek G., Scheithauer W., Steger G.G., Zielinski C., Unseld M. (2015). Nab-paclitaxel as second-line treatment in advanced biliary cancer. J. Clin. Oncol..

[29] Valle J.W., Lamarca A., Goyal L., Barriuso J., Zhu A.X. (2017). New Horizons for Precision Medicine in Biliary Tract Cancers. Cancer Discov..

[30] Arai Y., Totoki Y., Hosoda F., Shirota T., Hama N., Nakamura H., Ojima H., Furuta K., Shimada K., Okusaka T. (2014). Fibroblast growth factor receptor 2 tyrosine kinase fusions define a unique molecular subtype of cholangiocarcinoma. Hepatology.

[31] Wang L., Zhu H., Zhao Y., Pan Q., Mao A., Zhu W., Zhang N., Lin Z., Zhou J., Wang Y. (2020). Comprehensive molecular profiling of intrahepatic cholangiocarcinoma in the Chinese population and therapeutic experience. J. Transl. Med..

[32] Graham R.P., Barr Fritcher E.G., Pestova E., Schulz J., Sitailo L.A., Vasmatzis G., Murphy S.J., McWilliams R.R., Hart S.N., Halling K.C. (2014). Fibroblast growth factor receptor 2 translocations in intrahepatic cholangiocarcinoma. Hum. Pathol..

[33] Jain A., Borad M.J., Kelley R.K., Wang Y., Abdel-Wahab R., Meric-Bernstam F., Baggerly K.A., Kaseb A.O., Al-shamsi H.O., Ahn D.H. (2018). Cholangiocarcinoma With FGFR Genetic Aberrations: A Unique Clinical Phenotype. JCO Precis. Oncol..

[34] Abou-Alfa G.K., Sahai V., Hollebecque A., Vaccaro G., Melisi D., Al-Rajabi R., Paulson A.S., Borad M.J., Gallinson D., Murphy A.G. (2020). Pemigatinib for previously treated, locally advanced or metastatic cholangiocarcinoma: A multicentre, open-label, phase 2 study. Lancet Oncol..

[35] U.S. Food and Drug Administration: FDA Grants Accelerated Approval to Pemigatinib for Cholangiocarcinoma with an FGFR2 Rearrangement or Fusion. https://www.fda.gov/drugs/resources-information-approved-drugs/fda-grants-accelerated-approval-pemigatinib-cholangiocarcinoma-fgfr2-rearrangement-or-fusion.

[36] Javle M., Lowery M., Shroff R.T., Weiss K.H., Springfeld C., Borad M.J., Ramanathan R.K., Goyal L., Sadeghi S., Macarulla T. (2018). Phase II Study of BGJ398 in Patients With FGFR-Altered Advanced Cholangiocarcinoma. J. Clin. Oncol..

[37] Javle M., Kelley R.K., Roychowdhury S., Weiss K.H., Abou-Alfa G.K., Macarulla T., Sadeghi S., Waldschmidt D., Zhu A.X., Goyal L. (2018). Updated results from a phase II study of infigratinib (BGJ398), a selective pan-FGFR kinase inhibitor, in patients with previously treated advanced cholangiocarcinoma containing FGFR2 fusions. Ann. Oncol..

[38] Mazzaferro V., El-Rayes B.F., Droz Dit Busset M., Cotsoglou C., Harris W.P., Damjanov N., Masi G., Rimassa L., Personeni N., Braiteh F. (2019). Derazantinib (ARQ 087) in advanced or inoperable FGFR2 gene fusion-positive intrahepatic cholangiocarcinoma. Br. J. Cancer.

[39] Goyal L., Shi L., Liu L.Y., Fece de la Cruz F., Lennerz J.K., Raghavan S., Leschiner I., Elagina L., Siravegna G., Ng R.W.S. (2019). TAS-120 Overcomes Resistance to ATP-Competitive FGFR Inhibitors in Patients with FGFR2 Fusion–Positive Intrahepatic Cholangiocarcinoma. Cancer Discov..

[40] Tran B., Meric-Bernstam F., Arkenau H.T., Bahleda R., Kelley R.K., Hierro C., Ahn D., Zhu A., Javle M., Winkler R. (2018). Efficacy of TAS-120, an irreversible fibroblast growth factor receptor inhibitor (FGFRi), in patients with cholangiocarcinoma and FGFR pathway alterations previously treated with chemotherapy and other FGFRi’s. Ann. Oncol..

[41] Bahleda R., Italiano A., Hierro C., Mita A., Cervantes A., Chan N., Awad M., Calvo E., Moreno V., Govindan R. (2019). Multicenter Phase I Study of Erdafitinib (JNJ-42756493), Oral Pan-Fibroblast Growth Factor Receptor Inhibitor, in Patients with Advanced or Refractory Solid Tumors. Clin. Cancer Res..

[42] Vogel A., Sahai V., Hollebecque A., Vaccaro G., Melisi D., Al-Rajabi R., Paulson A.S., Borad M.J., Gallinson D., Murphy A.G. (2019). FIGHT-202: A phase II study of pemigatinib in patients (pts) with previously treated locally advanced or metastatic cholangiocarcinoma (CCA). Ann. Oncol..

[43] Liu S., Quarles L.D. (2007). How fibroblast growth factor 23 works. J. Am. Soc. Nephrol..

[44] Wöhrle S., Bonny O., Beluch N., Gaulis S., Stamm C., Scheibler M., Müller M., Kinzel B., Thuery A., Brueggen J. (2011). FGF receptors control vitamin D and phosphate homeostasis by mediating renal FGF-23 signaling and regulating FGF-23 expression in bone. J. Bone Min. Res..

[45] Wöhrle S., Henninger C., Bonny O., Thuery A., Beluch N., Hynes N.E., Guagnano V., Sellers W.R., Hofmann F., Kneissel M. (2013). Pharmacological inhibition of fibroblast growth factor (FGF) receptor signaling ameliorates FGF23-mediated hypophosphatemic rickets. J. Bone Min. Res..

[46] Mondesir J., Willekens C., Touat M., de Botton S. (2016). IDH1 and IDH2 mutations as novel therapeutic targets: Current perspectives. J. Blood Med..

[47] Raineri S., Mellor J. (2018). IDH1: Linking Metabolism and Epigenetics. Front. Genet..

[48] Zhao D.Y., Lim K.H. (2017). Current biologics for treatment of biliary tract cancers. J. Gastrointest Oncol..

[49] Boscoe A.N., Rolland C., Kelley R.K. (2019). Frequency and prognostic significance of isocitrate dehydrogenase 1 mutations in cholangiocarcinoma: A systematic literature review. J. Gastrointest Oncol..

[50] Abou-Alfa G.K., Macarulla T., Javle M.M., Kelley R.K., Lubner S.J., Adeva J., Cleary J.M., Catenacci D.V., Borad M.J., Bridgewater J. (2020). Ivosidenib in IDH1-mutant, chemotherapy-refractory cholangiocarcinoma (ClarIDHy): A multicentre, randomised, double-blind, placebo-controlled, phase 3 study. Lancet Oncol..

[51] Weil M.K., Chen A.P. (2011). PARP inhibitor treatment in ovarian and breast cancer. Curr. Probl. Cancer.

[52] Yap T.A., Plummer R., Azad N.S., Helleday T. (2019). The DNA Damaging Revolution: PARP Inhibitors and Beyond. Am. Soc. Clin. Oncol. Educ. Book.

[53] Chae H., Kim D., Yoo C., Kim K.-P., Jeong J.H., Chang H.-M., Lee S.S., Park D.H., Song T.J., Hwang S. (2019). Therapeutic relevance of targeted sequencing in management of patients with advanced biliary tract cancer: DNA damage repair gene mutations as a predictive biomarker. Eur. J. Cancer.

[54] Heeke A.L., Pishvaian M.J., Lynce F., Xiu J., Brody J.R., Chen W.-J., Baker T.M., Marshall J.L., Isaacs C. (2018). Prevalence of Homologous Recombination-Related Gene Mutations Across Multiple Cancer Types. JCO Precis. Oncol..

[55] Golan T., Raitses-Gurevich M., Kelley R.K., Bocobo A.G., Borgida A., Shroff R.T., Holter S., Gallinger S., Ahn D.H., Aderka D. (2017). Overall Survival and Clinical Characteristics of BRCA-Associated Cholangiocarcinoma: A Multicenter Retrospective Study. Oncologist.

[56] Jain A., Kwong L.N., Javle M. (2016). Genomic Profiling of Biliary Tract Cancers and Implications for Clinical Practice. Curr Treat Options Oncol..

[57] Spizzo G., Puccini A., Xiu J., Goldberg R.M., Grothey A., Shields A.F., Arora S.P., Khushmann M.d., Salem M.E., Battaglin F. (2020). Molecular profile of BRCA-mutated biliary tract cancers. ESMO Open.

[58] Sulkowski P.L., Corso C.D., Robinson N.D., Scanlon S.E., Purshouse K.R., Bai H., Liu Y., Sundaram R.K., Hegan D.C., Fons N.R. (2017). 2-Hydroxyglutarate produced by neomorphic IDH mutations suppresses homologous recombination and induces PARP inhibitor sensitivity. Sci. Transl. Med..

[59] Schvartzman J.M., Reuter V.P., Koche R.P., Thompson C.B. (2019). 2-hydroxyglutarate inhibits MyoD-mediated differentiation by preventing H3K9 demethylation. Proc. Natl. Acad. Sci. USA.

[60] Pignochino Y., Sarotto I., Peraldo-Neia C., Penachioni J.Y., Cavalloni G., Migliardi G., Casorzo L., Chiorino G., Risio M., Bardelli A. (2010). Targeting EGFR/HER2 pathways enhances the antiproliferative effect of gemcitabine in biliary tract and gallbladder carcinomas. BMC Cancer.

[61] Jensen L.H., Lindebjerg J., Ploen J., Hansen T.F., Jakobsen A. (2012). Phase II marker-driven trial of panitumumab and chemotherapy in KRAS wild-type biliary tract cancer. Ann. Oncol..

[62] Philip P.A., Mahoney M.R., Allmer C., Thomas J., Pitot H.C., Kim G., Donehower R.C., Fitch T., Picus J., Erlichman C. (2006). Phase II study of erlotinib in patients with advanced biliary cancer. J. Clin. Oncol..

[63] Chiorean E.G., Ramasubbaiah R., Yu M., Picus J., Bufill J.A., Tong Y., Coleman N., Johnston E.L., Currie C., Loehrer P.J. (2012). Phase II Trial of Erlotinib and Docetaxel in Advanced and Refractory Hepatocellular and Biliary Cancers: Hoosier Oncology Group GI06-101. Oncology.

[64] Gwak G.Y., Yoon J.H., Shin C.M., Ahn Y.J., Chung J.K., Kim Y.A., Kim T.Y., Lee H.S. (2005). Detection of response-predicting mutations in the kinase domain of the epidermal growth factor receptor gene in cholangiocarcinomas. J. Cancer Res. Clin. Oncol..

[65] Leone F., Cavalloni G., Pignochino Y., Sarotto I., Ferraris R., Piacibello W., Venesio T., Capussotti L., Risio M., Aglietta M. (2006). Somatic Mutations of Epidermal Growth Factor Receptor in Bile Duct and Gallbladder Carcinoma. Clin. Cancer Res..

[66] Guo Y., Feng K., Liu Y., Wu Z., Dai H., Yang Q., Wang Y., Jia H., Han W. (2018). Phase I Study of Chimeric Antigen Receptor–Modified T Cells in Patients with EGFR-Positive Advanced Biliary Tract Cancers. Clin. Cancer Res..

[67] Gutierrez C., Schiff R. (2011). HER2: Biology, detection, and clinical implications. Arch. Pathol. Lab. Med..

[68] Oh D.-Y., Bang Y.-J. (2020). HER2-targeted therapies—A role beyond breast cancer. Nat. Rev. Clin. Oncol..

[69] Javle M., Churi C., Kang H.C., Shroff R., Janku F., Surapaneni R., Zuo M., Barrera C., Alshamsi H., Krishnan S. (2015). HER2/neu-directed therapy for biliary tract cancer. J. Hematol. Oncol..

[70] Javle M.M., Hainsworth J.D., Swanton C., Burris H.A., Kurzrock R., Sweeney C., Meric-Bernstam F., Spigel D.R., Bose R., Guo S. (2017). Pertuzumab + trastuzumab for HER2-positive metastatic biliary cancer: Preliminary data from MyPathway. J. Clin. Oncol..

[71] Hyman D.M., Piha-Paul S.A., Won H., Rodon J., Saura C., Shapiro G.I., Juric D., Quinn D.I., Moreno V., Doger B. (2018). HER kinase inhibition in patients with HER2- and HER3-mutant cancers. Nature.

[72] Harding J., Cleary J., Shapiro G., Braña I., Moreno V., Quinn D., Borad M., Loi S., Spanggaard I., Stemmer S. (2019). Treating HER2-mutant advanced biliary tract cancer with neratinib: Benefits of HER2-directed targeted therapy in the phase 2 SUMMIT ‘basket’ trial. Ann. Oncol..

[73] Tan A.C., Oh D.-Y., Chao Y., Hsieh C.-Y., Chang W.-L., Isanto F., Chen Y.-C., McHale M., Lindmark B., Ng M.C.H. (2019). Efficacy and safety of varlitinib, a reversible pan-HER tyrosine kinase inhibitor, in combination with platinum-based regimens in biliary tract cancers: A pooled analysis from three phase I studies. J. Clin. Oncol..

[74] Ramanathan R.K., Belani C.P., Singh D.A., Tanaka M., Lenz H.J., Yen Y., Kindler H.L., Iqbal S., Longmate J., Mack P.C. (2009). A phase II study of lapatinib in patients with advanced biliary tree and hepatocellular cancer. Cancer Chemother. Pharmacol..

[75] Javle M.M., Oh D.-Y., Ikeda M., Yong W.-P., McIntyre N., Lindmark B., McHale M. (2020). Results from TreeTopp: A randomized phase II study of the efficacy and safety of varlitinib plus capecitabine versus placebo in second-line (2L) advanced or metastatic biliary tract cancer (BTC). J. Clin. Oncol..

[76] Feng K., Liu Y., Guo Y., Qiu J., Wu Z., Dai H., Yang Q., Wang Y., Han W. (2018). Phase I study of chimeric antigen receptor modified T cells in treating HER2-positive advanced biliary tract cancers and pancreatic cancers. Protein Cell..

[77] Tsurutani J., Iwata H., Krop I., Jänne P.A., Doi T., Takahashi S., Park H., Redfern C., Tamura K., Wise-Draper T.M. (2020). Targeting HER2 with Trastuzumab Deruxtecan: A Dose-Expansion, Phase I Study in Multiple Advanced Solid Tumors. Cancer Discov..

[78] Ohba A., Morizane C., Ueno M., Kobayashi S., Kawamoto Y., Komatsu Y., Ikeda M., Sasaki M., Okano N., Furuse J. (2020). Multicenter phase II study of trastuzumab deruxtecan (DS-8201) for HER2-positive unresectable or recurrent biliary tract cancer: HERB trial. J. Clin. Oncol..

[79] Zhang W., Liu H.T. (2002). MAPK signal pathways in the regulation of cell proliferation in mammalian cells. Cell Res..

[80] Schaider H., Sturm R.A. (2017). The evolving universe of BRAF mutations in melanoma. Br. J. Dermatol..

[81] Goeppert B., Frauenschuh L., Renner M., Roessler S., Stenzinger A., Klauschen F., Warth A., Vogel M.N., Mehrabi A., Hafezi M. (2014). BRAF V600E-specific immunohistochemistry reveals low mutation rates in biliary tract cancer and restriction to intrahepatic cholangiocarcinoma. Mod. Pathol..

[82] Hyman D.M., Puzanov I., Subbiah V., Faris J.E., Chau I., Blay J.-Y., Wolf J., Raje N.S., Diamond E.L., Hollebecque A. (2015). Vemurafenib in Multiple Nonmelanoma Cancers with BRAF V600 Mutations. N. Engl. J. Med..

[83] Bekaii-Saab T., Phelps M.A., Li X., Saji M., Goff L., Kauh J.S.W., O’Neil B.H., Balsom S., Balint C., Liersemann R. (2011). Multi-institutional phase II study of selumetinib in patients with metastatic biliary cancers. J. Clin. Oncol. Off. J. Am. Soc. Clin. Oncol..

[84] Kim R.D., McDonough S., El-Khoueiry A.B., Bekaii-Saab T.S., Stein S.M., Sahai V., Keogh G.P., Kim E.J., Baron A.D., Siegel A.B. (2020). Randomised phase II trial (SWOG S1310) of single agent MEK inhibitor trametinib Versus 5-fluorouracil or capecitabine in refractory advanced biliary cancer. Eur. J. Cancer.

[85] Finn R.S., Ahn D.H., Javle M.M., Tan B.R., Weekes C.D., Bendell J.C., Patnaik A., Khan G.N., Laheru D., Chavira R. (2018). Phase 1b investigation of the MEK inhibitor binimetinib in patients with advanced or metastatic biliary tract cancer. Investig. New Drugs.

[86] Kim J.W., Lee K.-H., Kim J.-W., Suh K.J., Nam A.-R., Bang J.-H., Bang Y.-J., Oh D.-Y. (2019). Enhanced antitumor effect of binimetinib in combination with capecitabine for biliary tract cancer patients with mutations in the RAS/RAF/MEK/ERK pathway: Phase Ib study. Br. J. Cancer.

[87] Eroglu Z., Ribas A. (2016). Combination therapy with BRAF and MEK inhibitors for melanoma: Latest evidence and place in therapy. Ther. Adv. Med. Oncol..

[88] Wainberg Z.A., Lassen U.N., Elez E., Italiano A., Curigliano G., De Braud F.G., Prager G., Greil R., Stein A., Fasolo A. (2019). Efficacy and safety of dabrafenib (D) and trametinib (T) in patients (pts) with BRAF V600E–mutated biliary tract cancer (BTC): A cohort of the ROAR basket trial. J. Clin. Oncol..

[89] Subbiah V., Lassen U., Élez E., Italiano A., Curigliano G., Javle M., de Braud F., Prager G.W., Greil R., Stein A. (2020). Dabrafenib plus trametinib in patients with BRAFV600E-mutated biliary tract cancer (ROAR): A phase 2, open-label, single-arm, multicentre basket trial. Lancet Oncol..

[90] Blumenschein G.R., Mills G.B., Gonzalez-Angulo A.M. (2012). Targeting the hepatocyte growth factor-cMET axis in cancer therapy. J. Clin. Oncol. Off. J. Am. Soc. Clin. Oncol..

[91] Miyamoto M., Ojima H., Iwasaki M., Shimizu H., Kokubu A., Hiraoka N., Kosuge T., Yoshikawa D., Kono T., Furukawa H. (2011). Prognostic significance of overexpression of c-Met oncoprotein in cholangiocarcinoma. Br. J. Cancer.

[92] Goyal L., Zheng H., Yurgelun M.B., Abrams T.A., Allen J.N., Cleary J.M., Knowles M., Regan E., Reardon A., Khachatryan A. (2017). A phase 2 and biomarker study of cabozantinib in patients with advanced cholangiocarcinoma. Cancer.

[93] Pant S., Saleh M., Bendell J., Infante J.R., Jones S., Kurkjian C.D., Moore K.M., Kazakin J., Abbadessa G., Wang Y. (2014). A phase I dose escalation study of oral c-MET inhibitor tivantinib (ARQ 197) in combination with gemcitabine in patients with solid tumors. Ann. Oncol..

[94] Corti F., Nichetti F., Raimondi A., Niger M., Prinzi N., Torchio M., Tamborini E., Perrone F., Pruneri G., Di Bartolomeo M. (2019). Targeting the PI3K/AKT/mTOR pathway in biliary tract cancers: A review of current evidences and future perspectives. Cancer Treat Rev..

[95] Roa I., de Toro G., Fernández F., Game A., Muñoz S., de Aretxabala X., Javle M. (2015). Inactivation of tumor suppressor gene pten in early and advanced gallbladder cancer. Diagn. Pathol..

[96] Turkes F., Carmichael J., Cunningham D., Starling N. (2019). Contemporary Tailored Oncology Treatment of Biliary Tract Cancers. Gastroenterol. Res. Pract..

[97] Wilson J.M., Kunnimalaiyaan S., Kunnimalaiyaan M., Gamblin T.C. (2015). Inhibition of the AKT pathway in cholangiocarcinoma by MK2206 reduces cellular viability via induction of apoptosis. Cancer Cell Int..

[98] RIZZO A., RICCI A.D., TOBER N., NIGRO M.C., MOSCA M., PALLONI A., ABBATI F., FREGA G., DE LORENZO S., TAVOLARI S. (2020). Second-line Treatment in Advanced Biliary Tract Cancer: Today and Tomorrow. Anticancer Res..

[99] Wu C.-E., Chen M.-H., Yeh C.-N. (2019). mTOR Inhibitors in Advanced Biliary Tract Cancers. Int. J. Mol. Sci..

[100] Ahn D.H., Li J., Wei L., Doyle A., Marshall J.L., Schaaf L.J., Phelps M.A., Villalona-Calero M.A., Bekaii-Saab T. (2015). Results of an abbreviated phase-II study with the Akt Inhibitor MK-2206 in Patients with Advanced Biliary Cancer. Sci. Rep..

[101] Buzzoni R., Pusceddu S., Bajetta E., De Braud F., Platania M., Iannacone C., Cantore M., Mambrini A., Bertolini A., Alabiso O. (2014). Activity and safety of RAD001 (everolimus) in patients affected by biliary tract cancer progressing after prior chemotherapy: A phase II ITMO study. Ann. Oncol..

[102] Leyva-Illades D., McMillin M., Quinn M., Demorrow S. (2012). Cholangiocarcinoma pathogenesis: Role of the tumor microenvironment. Transl. Gastrointest. Cancer.

[103] Simone V., Brunetti O., Lupo L., Testini M., Maiorano E., Simone M., Longo V., Rolfo C., Peeters M., Scarpa A. (2017). Targeting Angiogenesis in Biliary Tract Cancers: An Open Option. Int. J. Mol. Sci..

[104] Bengala C., Bertolini F., Malavasi N., Boni C., Aitini E., Dealis C., Zironi S., Depenni R., Fontana A., Del Giovane C. (2010). Sorafenib in patients with advanced biliary tract carcinoma: A phase II trial. Br. J. Cancer.

[105] Neuzillet C., Seitz J.-F., Fartoux L., Malka D., Lledo G., Tijeras-Raballand A., De Gramont A., Ronot M., Bouattour M., Dreyer C. (2015). Sunitinib as second-line treatment in patients with advanced intrahepatic cholangiocarcinoma (SUN-CK phase II trial): Safety, efficacy, and updated translational results. J. Clin. Oncol..

[106] Yi J.H., Thongprasert S., Lee J., Doval D.C., Park S.H., Park J.O., Park Y.S., Kang W.K., Lim H.Y. (2012). A phase II study of sunitinib as a second-line treatment in advanced biliary tract carcinoma: A multicentre, multinational study. Eur. J. Cancer.

[107] Zhao R., Zong H., Jin S., Zhong Q., Jiang M., Jin M., Li R., Jiang G. (2020). A phase II study of anlotinib with cediranib as a second-line treatment for patients with advanced biliary tract cancers (aBTCs). J. Clin. Oncol..

[108] Sun W., Patel A., Normolle D., Patel K., Ohr J., Lee J.J., Bahary N., Chu E., Streeter N., Drummond S. (2019). A phase 2 trial of regorafenib as a single agent in patients with chemotherapy-refractory, advanced, and metastatic biliary tract adenocarcinoma. Cancer.

[109] Kim R.D., Sanoff H.K., Poklepovic A.S., Soares H., Kim J., Lyu J., Liu Y., Nixon A.B., Kim D.W. (2020). A multi-institutional phase 2 trial of regorafenib in refractory advanced biliary tract cancer. Cancer.

[110] Demols A., Borbath I., Van den Eynde M., Houbiers G., Peeters M., Marechal R., Delaunoit T., Goemine J.C., Laurent S., Holbrechts S. (2020). Regorafenib after failure of gemcitabine and platinum-based chemotherapy for locally advanced/metastatic biliary tumors: REACHIN, a randomized, double-blind, phase II trial. Ann. Oncol..

[111] Ikeda M., Sasaki T., Morizane C., Mizuno N., Nagashima F., Shimizu S., Hayata N., Ikezawa H., Suzuki T., Nakajima R. (2017). 722P-A phase 2 study of lenvatinib monotherapy as second-line treatment in unresectable biliary tract cancer: Primary analysis results. Ann. Oncol..

[112] Amatu A., Sartore-Bianchi A., Siena S. (2016). *NTRK* gene fusions as novel targets of cancer therapy across multiple tumour types. Esmo Open.

[113] Cocco E., Scaltriti M., Drilon A. (2018). NTRK fusion-positive cancers and TRK inhibitor therapy. Nat. Rev. Clin. Oncol..

[114] Demols A., Rocq L., Charry M., De Nève N., Verrellen A., Ramadhan A., Van Campenhout C., De Clercq S., Salmon I., D’Haene N. (2020). NTRK gene fusions in biliary tract cancers. J. Clin. Oncol..

[115] Drilon A. (2019). TRK inhibitors in TRK fusion-positive cancers. Ann. Oncol..

[116] Drilon A., Laetsch T.W., Kummar S., DuBois S.G., Lassen U.N., Demetri G.D., Nathenson M., Doebele R.C., Farago A.F., Pappo A.S. (2018). Efficacy of Larotrectinib in TRK Fusion-Positive Cancers in Adults and Children. N. Engl. J. Med..

[117] Doebele R.C., Drilon A., Paz-Ares L., Siena S., Shaw A.T., Farago A.F., Blakely C.M., Seto T., Cho B.C., Tosi D. (2020). Entrectinib in patients with advanced or metastatic NTRK fusion-positive solid tumours: Integrated analysis of three phase 1-2 trials. Lancet Oncol..

[118] Rolfo C.D., De Braud F.G., Doebele R.C., Drilon A.E., Siena S., Patel M., Cho B.C., Liu S.V., Ahn M.-J., Chiu C.-H. (2020). Efficacy and safety of entrectinib in patients (pts) with NTRK-fusion positive (NTRK-fp) solid tumors: An updated integrated analysis. J. Clin. Oncol..

[119] Patel M., Siena S., Demetri G., Doebele R., Chae Y., Conkling P., Garrido-Laguna I., Longo F., Rolfo C., Sigal D. (2020). O-3 Efficacy and safety of entrectinib in NTRK fusion-positive gastrointestinal cancers: Updated integrated analysis of three clinical trials (STARTRK-2, STARTRK-1 and ALKA-372-001). Ann. Oncol..

[120] Wu C., Ma S. (2015). A selective review of robust variable selection with applications in bioinformatics. Brief Bioinform..

[121] Wu C., Zhou F., Ren J., Li X., Jiang Y., Ma S. (2019). A Selective Review of Multi-Level Omics Data Integration Using Variable Selection. High-Throughput.

[122] Jindal V. (2018). Immune checkpoint inhibitors in gastrointestinal malignancies. J. Gastrointest. Oncol..

[123] Havel J.J., Chowell D., Chan T.A. (2019). The evolving landscape of biomarkers for checkpoint inhibitor immunotherapy. Nat. Rev. Cancer.

[124] Ahn S., Lee J.-C., Shin D.W., Kim J., Hwang J.-H. (2020). High PD-L1 expression is associated with therapeutic response to pembrolizumab in patients with advanced biliary tract cancer. Sci. Rep..

[125] Marcus L., Lemery S.J., Keegan P., Pazdur R. (2019). FDA Approval Summary: Pembrolizumab for the treatment of microsatellite instability-high solid tumors. Clin. Cancer Res..

[126] Chan T.A., Yarchoan M., Jaffee E., Swanton C., Quezada S.A., Stenzinger A., Peters S. (2019). Development of tumor mutation burden as an immunotherapy biomarker: Utility for the oncology clinic. Ann. Oncol..

[127] Marabelle A., Le D.T., Ascierto P.A., Di Giacomo A.M., De Jesus-Acosta A., Delord J.P., Geva R., Gottfried M., Penel N., Hansen A.R. (2020). Efficacy of Pembrolizumab in Patients With Noncolorectal High Microsatellite Instability/Mismatch Repair-Deficient Cancer: Results From the Phase II KEYNOTE-158 Study. J. Clin. Oncol..

[128] Bang Y.J., Doi T., Braud F.D., Piha-Paul S., Hollebecque A., Razak A.R.A., Lin C.C., Ott P.A., He A.R., Yuan S.S. (2015). 525 Safety and efficacy of pembrolizumab (MK-3475) in patients (pts) with advanced biliary tract cancer: Interim results of KEYNOTE-028. Eur. J. Cancer.

[129] Bang Y.-J., Ueno M., Malka D., Chung H.C., Nagrial A., Kelley R.K., Piha-Paul S.A., Ros W., Italiano A., Nakagawa K. (2019). Pembrolizumab (pembro) for advanced biliary adenocarcinoma: Results from the KEYNOTE-028 (KN028) and KEYNOTE-158 (KN158) basket studies. J. Clin. Oncol..

[130] Ueno M., Ikeda M., Morizane C., Kobayashi S., Ohno I., Kondo S., Okano N., Kimura K., Asada S., Namba Y. (2019). Nivolumab alone or in combination with cisplatin plus gemcitabine in Japanese patients with unresectable or recurrent biliary tract cancer: A non-randomised, multicentre, open-label, phase 1 study. Lancet Gastroenterol. Hepatol..

[131] Kim R.D., Chung V., Alese O.B., El-Rayes B.F., Li D., Al-Toubah T.E., Schell M.J., Zhou J.-M., Mahipal A., Kim B.H. (2020). A Phase 2 Multi-institutional Study of Nivolumab for Patients With Advanced Refractory Biliary Tract Cancer. JAMA Oncol..

[132] Klein O., Kee D., Nagrial A., Markman B., Underhill C., Michael M., Jackett L., Lum C., Behren A., Palmer J. (2020). Evaluation of Combination Nivolumab and Ipilimumab Immunotherapy in Patients With Advanced Biliary Tract Cancers: Subgroup Analysis of a Phase 2 Nonrandomized Clinical Trial. JAMA Oncol..

[133] Jiao S., Xia W., Yamaguchi H., Wei Y., Chen M.-K., Hsu J.-M., Hsu J.L., Yu W.-H., Du Y., Lee H.-H. (2017). PARP Inhibitor Upregulates PD-L1 Expression and Enhances Cancer-Associated Immunosuppression. Clin. Cancer Res. Off. J. Am. Assoc. Cancer Res..

[134] Ioka T., Ueno M., Oh D.-Y., Fujiwara Y., Chen J.-S., Doki Y., Mizuno N., Park K., Asagi A., Hayama M. (2019). Evaluation of safety and tolerability of durvalumab (D) with or without tremelimumab (T) in patients (pts) with biliary tract cancer (BTC). J. Clin. Oncol..

[135] Hong T.S., Goyal L., Parikh A.R., Yeap B.Y., Ulysse C.A., Drapek L.C., Allen J.N., Clark J.W., Christopher B., Bolton C. (2020). A pilot study of durvalumab/tremelimumab (durva/treme) and radiation (XRT) for metastatic biliary tract cancer (mBTC): Preliminary safety and efficacy. J. Clin. Oncol..

[136] Ciciola P., Cascetta P., Bianco C., Formisano L., Bianco R. (2020). Combining Immune Checkpoint Inhibitors with Anti-Angiogenic Agents. J. Clin. Med..

[137] Arkenau H.-T., Martin-Liberal J., Calvo E., Penel N., Krebs M.G., Herbst R.S., Walgren R.A., Widau R.C., Mi G., Jin J. (2018). Ramucirumab Plus Pembrolizumab in Patients with Previously Treated Advanced or Metastatic Biliary Tract Cancer: Nonrandomized, Open-Label, Phase I Trial (JVDF). Oncology.

[138] Lin J., Shi W., Zhao S., Hu J., Hou Z., Yao M., Chrin G., Pan J., Hu K., Zhao L. (2018). Lenvatinib plus checkpoint inhibitors in patients (pts) with advanced intrahepatic cholangiocarcinoma (ICC): Preliminary data and correlation with next-generation sequencing. J. Clin. Oncol..

[139] Yarchoan M.C.L., Anders R.A., Anne Noonan A., Goff L.W., Goyal L., Lacy J., Li D., Patel A., He A.R., Abou-Alfa G. A multicenter randomized phase 2 trial of atezolizumab as monotherapy or in combination with cobimetinib in biliary tract cancers (BTCs): A NCI Experimental Therapeutics Clinical Trials Network (ETCTN) study. Proceedings of the 2020 American Association for Cancer Research Virtual Annual Meeting I.

[140] Block K.I., Block P.B., Gyllenhaal C. (2018). Integrative Treatment for Colorectal Cancer: A Comprehensive Approach. J. Altern Complement Med..

[141] Jentzsch V., Davis J.A.A., Djamgoz M.B.A. (2020). Pancreatic Cancer (PDAC): Introduction of Evidence-Based Complementary Measures into Integrative Clinical Management. Cancers.

[142] Abe K., Uwagawa T., Haruki K., Takano Y., Onda S., Sakamoto T., Gocho T., Yanaga K. (2018). Effects of ω-3 Fatty Acid Supplementation in Patients with Bile Duct or Pancreatic Cancer Undergoing Chemotherapy. Anticancer Res..

[143] Yu W.D., Ma Y., Flynn G., Muindi J.R., Kong R.X., Trump D.L., Johnson C.S. (2010). Calcitriol enhances gemcitabine anti-tumor activity in vitro and in vivo by promoting apoptosis in a human pancreatic carcinoma model system. Cell Cycle.

[144] Fountzilas C., Javle M., Tan W., Ma Y., Fetterly G., Iyer R., Johnson C. (2018). A phase 1, open-label, dose escalation study of intravenous paricalcitol in combination with gemcitabine in patients with advanced malignancies. Cancer.

